# Screening history of women with cervical cancer: a 6-year study in Aarhus, Denmark

**DOI:** 10.1038/sj.bjc.6604293

**Published:** 2008-03-11

**Authors:** O Ingemann-Hansen, M Lidang, I Niemann, J Dinesen, U Baandrup, H Svanholm, L K Petersen

**Affiliations:** 1 Institute of Pathology, Aarhus University Hospital, Norrebrogade 44, 8000 Aarhus C, Denmark; 2 Institute of Pathology, Herlev Hospital, Herlev Ringvej 75, 2730 Herlev, Denmark; 3 Department of Gynaecology, Aarhus University Hospital, Brendstrupgaardsvej 100, 8200 Aarhus N, Denmark; 4 Institute of Pathology, Vendsyssel Hospital, Bispensgade 37, 9800 Hjorring, Denmark; 5 Institute of Pathology, Regionshospital Randers, Skovlyvej 1, 8900 Randers, Denmark

**Keywords:** cervical cancer, screening history, cervical smear, prevention

## Abstract

To identify possible weaknesses in cervical screening in Aarhus County, 10 years after the programme was introduced, screening histories were examined. A major problem for the screening programme was that 31% of women were never screened and 61% under-screened, the latter group being significantly dominated by older women and high-stage tumours.

The incidence of cervical cancer in Danish women is among the highest worldwide (14.5 per 100 000 women) (Danish National Board of Health, 2007). In Aarhus County, cytological screening started in March 1989. Every third year, all women aged 23–59 years receive a personal invitation to have a Papanicolaou (PAP) smear taken free of charge by their general practitioner. If the woman does not attend, she receives a re-invitation after 6 months.

The aim of this study was to identify possible weaknesses in the screening process 10 years after the programme was initiated. Since every case of cervical cancer arising in a well-organised screening programme must be considered a possible failure, we wished to focus on the screening history of women with cervical cancer diagnosed more than 10 years after screening was introduced.

## MATERIALS AND METHODS

The study population consisted of 286 patients identified electronically with histologically verified cervical cancer during the period 1 January 1997 to 30 October 2002 in Aarhus County (322 209 female inhabitants in 2000) by cervical biopsy, cervical abrades, conisation or hysterectomy specimens. The results of all previous cervical smears (CS) and histological samples from each patient were subsequently found in the national pathology databank, and registered according to the diagnostic categories of WHO. Information on age and tumour stage at diagnosis (ad modem FIGO) was obtained from medical records. Patients were distinguished according to whether they were diagnosed by the screening programme or because of symptoms such as abnormal vaginal bleeding, vaginal discharge or pain.

A ‘trigger smear’ was defined for each patient as the smear leading to further examination. For the majority of patients with cervical cancer, the trigger smear, or more rarely a histological sample, started a ‘diagnostic period’ that continued for up to 5 months. To evaluate the previous screening process in each woman, we defined an ‘intervention period’ from 5 to 47 months before the diagnosis, during which a possible preinvasive stage or earlier cancer diagnosis should ideally have been detected. An intervention period of 42 months was chosen to give the women a 6-month period to respond to an invitation, as used previously (Sung *et al*, 2000). In the collected data, patients were scored into six different groups based on the screening history:

1. ‘Failure to screen in the intervention period’ included women whose last smear was taken outside the recommended screening interval;
2. ‘Never screened’ included women who were never screened previously;
3. ‘Failure in detection’ included women having a CS in the intervention period reported as normal. This group could be the false-negative results of PAP screening (either due to an inadequate or poor smear or failure in reading the smear), as one would expect at least a precancerous diagnosis within 3.5 years before an invasive cancer;
4. ‘Abnormal CS and adequate follow-up’ included women having an abnormal smear result in the intervention period and receiving adequate follow-up afterward;
5. ‘Failure in follow-up’ included women with abnormal CS in the intervention period and no or delayed follow-up before the trigger CS (i.e., a trigger delay). The registered trigger smear could either be upon a new invitation, after symptoms or the result of an inadequate follow-up guidance;
6. ‘Other screening history’ included patients outside the other groups and consisted of women already in a follow-up (e.g., after conisation).

A PAP smear was defined as normal if no further diagnostic procedures were suggested and abnormal if the cytologist recommended a follow-up. Non-parametric statistics using SPSS 9.0 and prevalence proportion ratio (PPR) with 95% confidence interval (95% CI) were determined.

## RESULTS

The diagnosis of cervical cancer resulted from referral because of symptoms in 58% cases, the most frequent being bleeding (48%). Women over 60 years of age had a PPR of 2.1 (95% CI: 1.8–2.5) for being diagnosed because of symptoms compared with women less than 60 years; the latter were more likely to have microscopic/local disease than older women (PPR 1.6; 95% CI: 1.3–2.0).

As shown in Table 1, the majority (173) of patients had no CS during the intervention period (groups 1 and 2), and 107 (62%) of these patients were of an appropriate age for participating in the screening procedure when diagnosed with cancer. Failure to screen was associated more commonly with age of over 60 years (PPR 1.7; 95% CI: 1.2–2.3) and with high-stage cancer (PPR 2.0; 95% CI: 1.4–2.9). Inflammation was an associated cytological diagnosis in the intervention CS among 38% of group 3. Going further back in screening histories, as many as 75% women had once been diagnosed with inflammation. Comparing group 3 with the other groups of ‘well-screened’ patients (groups 4–6) demonstrated a significant incidence of previous inflammation (PPR 1.6; 95% CI: 1.1–2.2).

## DISCUSSION

The most important finding of this study is the disappointing fact that despite organised screening since 1989, only 42% of cervical cancers were diagnosed within the screening programme in Aarhus County (Denmark).

The facts that 23% of women with cervical cancer never had a PAP smear and 61% of the cases were non-adherent to screening (i.e., latest CS more than 3.5 years before the trigger smear) represent major problems for the programme. Similar problems have been reported previously, with proportions non-adherent to the screening programme being 53% (Sung *et al*, 2000), 54% (Spence *et al*, 2007) and 56% (Leyden *et al*, 2005), and Spence *et al* (2007) further estimated that 42% were never screened. Our findings demonstrate, not surprisingly, that older women are associated with non-adherence and higher stages of cancer. However, about two-thirds of non-adherent women were the focus of the screening programme, so our results definitively confirm that increasing participation in the screening programme should be given high priority, to reduce the incidence of cervical cancer (Lynge *et al*, 2006).

We were unable to differentiate between rapid-onset cancers and ‘true’ false-negative results. The existence of rapid-onset cancers is debatable, but we considered that a precancerous smear diagnosis was to be expected in the previous 3.5-year period (Wain *et al*, 1992). Thus, it seems most likely that the women in group 3 represent 20% false-negative smears due to sampling error or diagnostic error. A similar false-negative rate has been reported recently (Spence *et al*, 2007) and in an earlier Danish study (Ingeholm and Glenthoj, 1996). We found a significant difference in favour of PAP smears with inflammation in group 3, an obscuring factor that may lead to misinterpretation and underlines the importance of high smear quality (Skehan *et al*, 1990; Sherman and Kelly, 1992; Lynge *et al*, 1993; Lyall and Duncan, 1995).

Our findings indicate that efforts should not only be made to motivate women to attend the screening, but also in the organisation of cervical screening. Including women aged 60–69 years in the screening programme will potentially increase the detection of cervical cancer by 10%. A study on the false-negative PAP smears has been launched and may identify subgroups of special interest.

## Figures and Tables

**Table 1:** Age, clinical stage, histological type of cervical cancer and previous inflammation according to PAP smear histories of 286 women in Aarhus County (1997–2002)^a^

	**Group division by screening history**							**Bivariate analyses**		
	**Group I (*n*=108)**	**Group II (*n*=65)**	**Group III (*n*=57)**	**Group IV (*n*=21)**	**Group V (*n*=13)**	**Group VI (*n*=22)**		**I *vs* II**	**I *vs* III–VI**	**III *vs* IV–VI**
**Variable**	**Failure to screen**	**Never screened**	**Failure in detection**	**Adequate follow-up**	**Inadequate follow-up**	**Other**	**Total (*n*=286)**	**PPR (95% CI)**	**PPR (95% CI)**	**PPR (95% CI)**
% of all	38	23	20	7	5	7	100			
Mean age	49.6	59.2	41.7	36.0	41.3	49.3	49.5			
										
*Age*										
<40	40 (37)	6 (9)	27 (47)	15 (71)	6 (46)	4 (18)	100 (35)	1	1	1
40–59	44 (41)	17 (26)	26 (46)	6 (29)	6 (46)	13 (59)	112 (39)	0.8 (0.7–1.0)	1.1 (0.8–1.5)	1.0 (0.7–1.4)
⩾60	24 (22)	42 (65)	4 (7)	0	1 (8)	5 (23)	76 (26)	0.4 (0.3–0.6)	1.7 (1.2–2.3)	0.8 (0.4–1.7)
										
*Stage*										
I.A	31 (29)	6 (9)	26 (46)	11 (52)	5 (38)	8 (36)	87 (30)	1	1	1
I.B–II.A	52 (48)	31 (48)	30 (53)	9 (43)	6 (46)	5 (23)	133 (47)	0.8 (0.6–0.9)	1.3 (1.0–1.9)	1.2 (0.8–1.7)
II.B–IV	21 (19)	25 (38)	1 (1)	1 (5)	2 (16)	2 (9)	52 (18)	0.5 (0.4–0.8)	2.0 (1.4–2.9)	0.3 (0.1–2.0)
Unknown	4 (4)	3 (5)	0	0	0	7 (32)	14 (5)	—	—	—
										
*Histological*										
Squamous	92 (85)	55 (85)	45 (79)	17 (81)	11 (85)	13 (59)	233 (81)	1.0 (0.7–1.4)	1.4 (0.9–2.1)	1.2 (0.7–1.9)
Non-squamous	16 (15)	10 (15)	12 (21)	4 (19)	2 (15)	9 (41)	53 (19)	1	1	1
										
*Cytological*										
Inflammation	57 (53)	—	42 (75)	10 (48)	6 (46)	10 (56)	125 (43)	—	—	1.6 (1.1–2.2)

Abbreviations: CI=confidence interval; PAP=Papanicolaou; PPR=prevalence proportion ratio.

a Values are given in years and as *n* (%).
